# Perisylvian white matter connectivity in the human right hemisphere

**DOI:** 10.1186/1471-2202-10-15

**Published:** 2009-03-03

**Authors:** Alireza Gharabaghi, Frank Kunath, Michael Erb, Ralf Saur, Stefan Heckl, Marcos Tatagiba, Wolfgang Grodd, Hans-Otto Karnath

**Affiliations:** 1 Functional and Cognitive Neurosurgery Unit, Department of Neurosurgery and Werner Reichardt Centre for Integrative Neuroscience, University of Tübingen, 72076 Tübingen, Germany; 2 Section Experimental NMR of the CNS, Department of Neuroradiology, University of Tübingen, 72076 Tübingen, Germany; 3 Section of Neuropsychology, Center of Neurology, Hertie-Institute for Clinical Brain Research, University of Tübingen, 72076 Tübingen, Germany

## Abstract

**Background:**

By using diffusion tensor magnetic resonance imaging (DTI) and subsequent tractography, a perisylvian language network in the human left hemisphere recently has been identified connecting Brocas's and Wernicke's areas directly (arcuate fasciculus) and indirectly by a pathway through the inferior parietal cortex.

**Results:**

Applying DTI tractography in the present study, we found a similar three-way pathway in the right hemisphere of 12 healthy individuals: a direct connection between the superior temporal and lateral frontal cortex running in parallel with an indirect connection. The latter composed of a posterior segment connecting the superior temporal with the inferior parietal cortex and an anterior segment running from the inferior parietal to the lateral frontal cortex.

**Conclusion:**

The present DTI findings suggest that the perisylvian inferior parietal, superior temporal, and lateral frontal corticies are tightly connected not only in the human left but also in the human right hemisphere.

## Background

It is well known that some brain functions are asymmetrically represented in the human brain. While an elaborate representation for language has evolved in the human left hemisphere, a neural system involved in spatial orienting and exploration is dominantly represented in the right hemisphere. Recently, Catani and co-workers [1] re-explored the putative pathways between the perisylvian language areas of the human left hemisphere using diffusion tensor imaging (DTI) tractography. DTI is a technique to identify restricted motion of water molecules, which can be used to assess the in vivo connectivity of human brain areas by quantifying the diffusion characteristics in the white matter [2]. In each magnetic resonance imaging (MRI) voxel, the direction of the fastest diffusion represents the dominant direction of the white matter tracts [3]. Thereby it is possible to obtain estimates of fibre orientation [4], leading to three-dimensional illustrations of white matter pathways [5-8].

Using this technique Catani and colleagues [1] could visualize a direct connection between the lateral frontal and the superior temporal cortex, representing the arcuate fasciculus between Broca's and Wernicke's language areas. However, in addition to this classical direct pathway, the authors also found an indirect pathway between these two areas running lateral to the arcuate fasciculus and passing through the inferior parietal cortex. This indirect pathway consisted of a posterior segment connecting superior temporal cortex (Wernicke's area) with the inferior parietal cortex and an anterior segment connecting the inferior parietal with the lateral frontal cortex (Broca's area). Catani et al. [1] thus speculated that this pathway might serve for semantically based language functions such as auditory comprehension and vocalization of semantic content.

The present study used DTI tractography to investigate the connectivity between the same perisylvian areas in the healthy human right hemisphere. We were interested to study the connecting fiber bundels between the superior temporal, inferior parietal, and the lateral frontal cortices since these distant, perisylvian regions have repeatedly been described to provoke the same disorder in the case of brain damage, namely spatial neglect [9-15].

## Results

**Figure 1:**
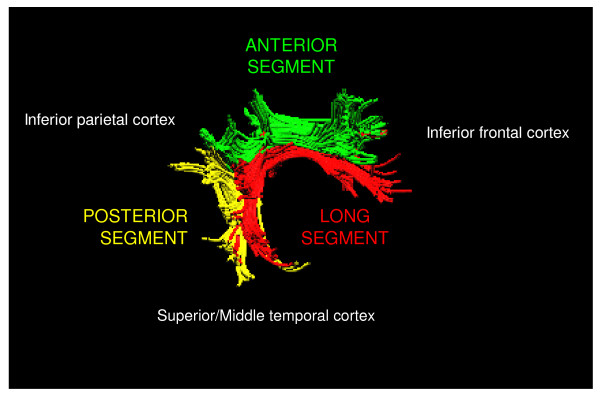
**Averaged tractography reconstruction by using a two-region of interest approach**. It shows a three-way connection between the superior temporal, inferior parietal, and the lateral frontal cortex. The direct connection between the superior temporal and lateral frontal cortex is shown in red. The posterior segment of the indirect connection, running from the superior temporal to the inferior parietal cortex is shown in yellow. The anterior segment of the indirect connection, running from the inferior parietal to the lateral frontal cortex is shown in green.

Twelve right-handed male subjects without neurological deficits were investigated. Figure 1 shows the tractography reconstruction using a two-region of interest approach obtained from the average DTI data set of the twelve examined individuals. The averaged tractography shows a three-way connection between the superior temporal, inferior parietal, and lateral frontal cortex in the human right hemisphere. The three segments are color-coded as follows: The direct connection between the superior temporal and lateral frontal cortex is shown in red. The posterior segment of the indirect connection, running from the superior temporal to the inferior parietal cortex is shown in yellow. The anterior segment of the indirect connection, running from the inferior parietal to the lateral frontal cortex is shown in green.

**Figure 2:**
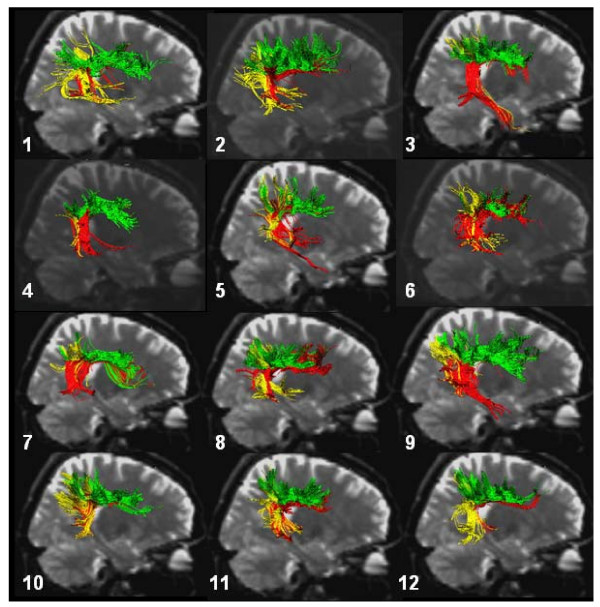
**Reconstructed direct and indirect pathways (using a two-region of interest approach) in each of the 12 healthy subjects**. Reconstructions were superimposed on sagittal b0 data. Color coding as in Figure 1.

Figure 2 shows the individual DTI tractography results of each of the 12 subjects using the same colour-coding schema as Figure 1. In fact, the three-way connection was present in all cases, although with individual variations. We found the long (red) segment more prominent in some individuals than in others. While subjects 3, 4, 5, 6, 7, 8, and 9 showed a strong portion of this segment, it was less prominent in subjects 1, 2, 10, 11, and 12 (Fig. 2). Moreover, in subjects 3, 5, 6, and 9, the projections into the temporal lobe extended farther than in subjects 1, 2, 4, 7, 8, 10, 11, and 12.

## Discussion

This work investigated the neural connectivity of the perisylvian areas in the human right hemisphere. Comparable with the anatomical findings in the human left hemisphere [1], we found an indirect connection between lateral frontal and superior temporal cortices composed of a posterior segment connecting the superior temporal with the inferior parietal cortex and an anterior segment running from the inferior parietal to the lateral frontal cortex. Running partly in parallel with the fibers from the indirect connection, we also found a direct connection between the superior temporal and the lateral frontal cortex representing the classical arcuate fasciculus. Historically, the arcuate fasciculus (AF) and the superior longitudinal fasciculus (SLF) have been considered a single entity [16,17]. In contrast to these classical descriptions, recent DTI studies in humans [18] and non-human primates [19] could identify different fibre bundles. Makris and co-workers [18] identified four major subcomponents of the SLF in the human brain with the arcuate fasciculus as its forth subdivision (SLF IV). It stems from the superior temporal gyrus, arches around the caudal end of the Sylvian fissure, and extends to the lateral frontal cortex [18].

While we were conducting the present investigation, Catani and colleagues published a study [20] in which they also had analysed the perisylvian connectivity in the human right hemisphere. In line with the present findings, they found an indirect connection with a posterior segment connecting the superior temporal with the inferior parietal cortex and an anterior segment running from the inferior parietal to the lateral frontal cortex. In contrast, they found the long, direct segment between the superior temporal and the lateral frontal cortices only in about 40% of their individuals, while this segment was present in all of our subjects. Again in line with Catani et al.'s [20] findings, the present analysis also observed between-subject differences with respect to the long, direct segment. While some of the subjects showed a strong portion of this segment, it was less prominent in others. However, when we calculated the average template from the DTI data sets of all our 12 subjects, we revealed a tractography reconstruction showing a three-way connection between the superior temporal, inferior parietal, and the lateral frontal cortex in the right hemisphere, similar to the one that Catani et al. [1] had reported for the human left hemisphere.

Recent studies compared volume, bundle density, or location of the AF and/or SLF between the human left and right hemispheres [18,21,22]. Unlike the recent findings of Catani et al. [20] these studies only reported slight numerical but insignificant differences comparing the corresponding fibre tracts between the two hemispheres [18,21,22]. Except for a leftward asymmetry of subcomponent III of the SLF [18], volume, bundle density, and location of the left- and right-sided fiber bundles appeared grossly symmetrical. However, discrepant observations also have been reported [23,24]. Vernooij et al. [24] found a clear leftward asymmetry for the AF in about 80% of their subjects. However, they found this asymmetry irrespective of the lateralisation of language representation, while Catani and co-workers [20] found the leftward asymmetry negatively correlated with the performance in a verbal word-list learning task. Possible reasons for the discrepancies between these studies (including our present study) can be attributed to differences in fiber tracking methods (deterministic, probabilistic), in the choice of the seeding ROIs, and/or the composition of subject samples. Further research is needed to clarify these issues.

## Conclusion

In summary, the present DTI findings suggest that the perisylvian inferior parietal, superior temporal, and lateral frontal corticies are tightly connected not only in the human left but also in the human right hemisphere.

## Methods

Twelve right-handed male subjects (median age = 23.5 years, range 19–32 years) without neurological deficits were investigated. All subjects gave their informed consent to participate in the study which was performed in accordance with the ethical standards established by the 1964 Declaration of Helsinki. MR images were acquired on a 1.5 T scanner (Sonata, Siemens, Erlangen). The DTI data were acquired along 6 non-collinear gradient directions with an EPI sequence with 60 axial slices covering the brain from the pons to the vertex (TR = 7300 ms, TE = 80 ms, flip-angle = 90°, b-value = 800 s/mm^2^, 2.5 mm slice thickness, no gap, matrix = 128 × 128 pixel, FOV = 238 × 238 mm^2^). Measurements were repeated 4 times to increase the signal-to-noise ratio. All data sets were re-aligned for motion correction and averaged with SPM2 (Statistical Parametric Mapping, London) program package using the b0-images to estimate the transformation parameters. Then each of the diffusion weighted mean images was co-registered and resliced to mean unweighted image (b0) in order to reduce differences in positions. The resulting mean data sets were normalized to the MNI-EPI template included in SPM2.

For each individual data set the normalized eigenvector map, FA map, colour map, and tensor trace map were calculated with DTIstudio (Jiang/Mori, John Hopkins University, Baltimore) by using the equivalent rotated gradient scheme. In-house software (M. Erb, Section Experimental NMR of the CNS) allowed splitting up individual eigenvector and colormap vector files to the x-, y-, and z-components in SPM2. This was repeated in each subject. Mean x-, y- and z-components were calculated based on the individual data sets. Finally, the vector data sets for eigenvector and color maps were combined from the mean x-, y-, and z-components.

**Figure 3:**
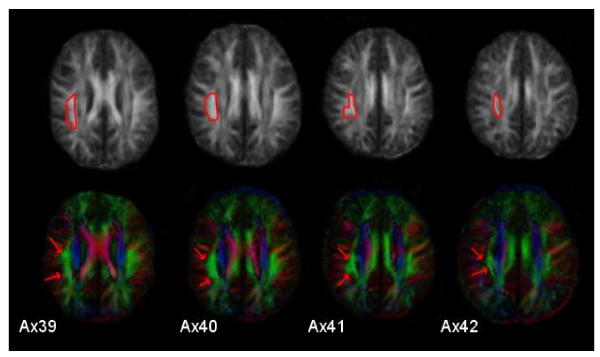
**Demarcation of the region of interest around the arcuate fasciculus in the right hemisphere**. The lower row presents axial color fibre orientation maps from a single subject (Talairach z = 39–42). The standard color coding for fiber orientation is used (red: lateral-to-lateral; green: anterior-posteriorly or vice versa; blue: superior-inferiorly or vice versa). Red arrows indicate the extension of the homologues of the arcuate fasciculus. According to this color fiber orientation map, a region of interest (encircled in red) is defined in the fractional anisotropy image (upper row).

Fiber tracking was processed using DTIStudio. The calculation for each of the 12 individuals (Fig. 2) was based on the normalized eigenvector map and FA map by following the direction of the main diffusion (FA threshold = 0.25, abortion angle = 69°). The averaged tractography reconstruction (Fig. 1) was processed for the mean data set based on the mean eigenvector map and FA map by following the direction of the main diffusion (FA threshold = 0.25, abortion angle = 69°). For every data set the same criteria were used. Following the procedure used by Catani et al. [1], in a first step, ROIs on the right hemisphere were localized on the fractional anisotropy images in z = 39–42 (Figure 3). To investigate detailed dissections, a two-ROI approach was used in order to separate different sets of fibres [1]. This approach defines two spatially distinguished areas in the fractional anisotropy volume, thereby, visualizing all fibres passing through both using an "AND" condition. This technique does not constrain tracts to start and end within the defined areas, only to pass through them [1]. To attach different colours to the different sets of fibres, in-house software (R. Saur, Section Experimental NMR of the CNS) was used. Finally, 3D visualization was performed on the b0-images using BrainVoyager QX (Goebel, Brain Innovation, Maastricht).

## Authors' contributions

HOK and AG proposed the general research question. AG, MT, WG, and HOK designed the experiment. AG, FK, ME, RS, and SH accomplished data collection, analyzed the data and contributed the methods and results sections. HOK and AG drafted the initial version of the manuscript. All authors read and approved the final manuscript.
